# Identification and diagnostic potential of pyroptosis-related genes in endometriosis: A novel bioinformatics analysis and validation

**DOI:** 10.1371/journal.pone.0350751

**Published:** 2026-06-09

**Authors:** Li Wang, Piaopiao Teng, Jiawen Chen, Caiyun Ding, Xianchen Luo, Chang Su, Guantai Ni, Yuanyuan Lyu, Jin Ding

**Affiliations:** 1 Department of Obstetrics and Gynecology, First Affiliated Hospital of Wannan Medical University, Wuhu, Anhui, Peoples’s Republic of China; 2 Modern Health and Wellness Industry College, Anhui Sanlian University, Hefei, Anhui, Peoples’s Republic of China; 3 Department of Basic Medicine, Wannan Medical University, Wuhu, Anhui, Peoples’s Republic of China; 4 Department of Obstetrics and Gynecology, Southeast University Zhongda Hospital, Nanjing, Peoples’s Republic of China; Noorda College of Osteopathic Medicine, UNITED STATES OF AMERICA

## Abstract

**Background:**

Endometriosis (EMs) is a chronic inflammatory disease characterized by ectopic endometrial growth. This study aimed to identify and analyze potential signatures of pyroptosis-related genes in EMs.

**Methods:**

We conducted a comprehensive bioinformatics analysis using transcriptomic datasets from the GEO database to identify pyroptosis-related differentially expressed genes (PRDEGs) in endometriosis. Gene Set Enrichment Analysis (GSEA), Gene Set Variation Analysis (GSVA), Weighted Gene Co-expression Network Analysis (WGCNA), and Protein-Protein Interaction (PPI) network construction were applied to explore the functional relevance of PRDEGs. A candidate gene signature was constructed using Least Absolute Shrinkage and Selection Operator (LASSO) regression based on pyroptosis scores, and its predictive performance was evaluated in an independent dataset. The expression of key PRDEGs was validated by RT-qPCR in eutopic and ectopic endometrial tissue samples from patients (n = 10 each).

**Results:**

Based on the pyroptosis score, endometriosis samples were divided into high- and low-score groups, with a significant difference in score distribution between the two groups. This score was primarily used to characterize the pyroptosis-related stratification features within the samples. Further screening of differentially expressed genes identified five candidate diagnostic-related genes (*KIF13B, BAG6, MYO5A, HEATR2,* and *AK055981*). The model constructed using these genes showed moderate discriminatory ability in an independent dataset. RT-qPCR results confirmed differential expression of *KIF13B, BAG6, MYO5A,* and *HEATR2* between ectopic and normal endometrial tissues, and several IL-17 pathway‑related genes exhibited consistent trends.

**Conclusions:**

This study suggests a potential role for pyroptosis in endometriosis and identifies a candidate gene signature. These findings may provide new clues for understanding inflammation- and cell death-related mechanisms in endometriosis and serve as a reference for future studies conducted in larger cohorts and under more rigorous validation frameworks.

## 1. Background

Endometriosis is defined by the presence of endometrium-like epithelium and/or stroma outside the uterus [1]. It affects approximately 10% of women of childbearing age and significantly impairs their quality of life. and results in symptoms such as pain and infertility [2]. Endometriosis is characterized as an inflammatory condition, wherein the presence of ectopic lesions can precipitate pelvic inflammation, thereby facilitating the further development of these ectopic endometrial lesions. The recurrent inflammatory responses associated with this condition result in an abnormal elevation of inflammatory cytokines, which are essential for the attachment, proliferation, and invasion of ectopic endometrial lesions [3]. However, the fundamental mechanisms underlying endometriosis remain poorly understood, and further research is required to elucidate its pathogenesis.

Pyroptosis is an inflammatory form of programmed cell death initiated by a variety of disease-causing factors. It has received increasing attention because of the association with innate immunity and disease [4,5]. It has been established that pyroptosis is induced within the stromal compartments of endometriotic lesions by bufalin. This induction is associated with increased activity of IL-1β and caspase-1 in stromal cells [6]. The release of prostaglandin E2 (PGE2) as a consequence of pyroptosis has been implicated in the advancement of this disease by influencing cellular migration [7]. Additionally, TRIM24 may play a role in the progression of endometriosis through the NLRP3/caspase-1/interleukin-1 (IL-1) mediated pyroptotic pathway, potentially via the ubiquitination of NLRP3 [8]. The findings of these studies indicate that pyroptosis plays a role in the pathogenesis of endometriosis.

The growing prevalence and utilization of gene chip sequencing technology have rendered microarray analysis a valuable and innovative approach for the identification of genes associated with susceptibility to endometriosis. The present study aimed to identify pyroptosis-related genes (PRGs) in endometriosis patients through bioinformatics analysis of transcriptome sequencing data, thereby providing new insights into the molecular mechanisms of this condition.

## 2. Methods

### 2.1. Data collection and analysis of PRDEGs

Endometriosis-related transcriptomic datasets (GSE7305, GSE7307, GSE11691) and the validation dataset (GSE25628) were obtained from the GEO database using the GEOquery package in R (v4.2.0). These datasets included Homo sapiens endometrial samples. Batch effects were corrected with the sva package, yielding a combined dataset of 37 endometriosis and 42 normal samples.

To obtain a comprehensive set of pyroptosis-related genes (PRGs), we searched the GeneCards and Gene Set Enrichment Analysis (GSEA) databases using the keyword “pyroptosis”. From the GeneCards database, only entries with the “Protein Coding” category were retained. For the GSEA database, we retrieved functionally annotated gene sets associated with pyroptosis. After merging the genes from both sources and removing duplicates, a final list of 338 candidate PRGs was compiled (S1 Table).

Differentially expressed genes (DEGs) between endometriosis and normal samples were identified using DESeq2 (|logFC| > 1, adj.p < 0.05). Pyroptosis-related DEGs (PRDEGs) were identified by intersecting DEGs with a curated pyroptosis gene list (Venn diagram). Visualization included volcano plots, heatmaps, chromosome localization, and correlation plots (ggplot2, pheatmap, RCircos). Gene Ontology (GO) and Kyoto Encyclopedia of Genes and Genomes (KEGG) enrichment analyses of PRDEGs were performed using clusterProfiler.

### 2.2. Construction and validation of a candidate gene signature for endometriosis

Pyroptosis scores for endometriosis patients were calculated using the single-sample Gene Set Enrichment Analysis (ssGSEA) algorithm and used to classify patients into high- and low-expression groups based on the median score. A candidate signature was built using the randomForest package in R, followed by Least Absolute Shrinkage and Selection Operator (LASSO) regression analysis with the glmnet package (set.seed(500), family = “cox”) to identify key diagnostic features. A nomogram was created with the rms package to visualize the model. The signature’s predictive performance was assessed in the independent validation dataset GSE25628 using decision curve analysis (DCA) plots generated with the ggDCA package.

### 2.3. Gene set enrichment analysis (GSEA) and gene set variation analysis (GSVA)

Gene Set Enrichment Analysis (GSEA) and Gene Set Variation Analysis (GSVA) were performed using the clusterProfiler R package on endometriosis samples. Genes were ranked by phenotypic correlation, and the hallmark gene set (MSigDB, v7.4) was used for pathway enrichment. Differential pathway enrichment between high and low pyroptosis expression groups was assessed (p < 0.05).

### 2.4. Weighted gene co-expression network analysis (WGCNA)

Weighted Gene Co-expression Network Analysis (WGCNA) was performed in R to identify gene modules correlated with pyroptosis expression. The top two most correlated modules were selected, and their genes were intersected with DEGs from high and low pyroptosis groups (Venn diagrams). The resulting overlapping genes were designated as key module genes (KMGs).

### 2.5. PPI network and regulatory networks

A PPI network for differentially expressed KMGs was constructed using the STRING database with a minimum interaction score of 0.150 (low confidence). Hub genes were identified using three algorithms in the cytoHubba plugin of Cytoscape: Maximum Neighborhood Component (MNC), Degree, and Maximal Clique Centrality (MCC). The intersection of hub genes from the three algorithms was visualized with a Venn diagram, and the resulting genes were defined as pyroptosis-related hub genes. Regulatory networks, including competing endogenous RNA (ceRNA), transcription factor-mRNA (TF-mRNA), mRNA-RNA binding protein (mRNA-RBP), and drug-mRNA interactions, were constructed and visualized in Cytoscape using the StarBase, ChIPBase, and Comparative Toxicogenomics Database (CTD).

### 2.6. Construction of disease subtypes of endometriosis

Consensus Clustering (CC), a resampling-based methodology, was employed to ascertain the membership of individual data points and their corresponding subgroup classifications, while also evaluating the validity of the identified clusters. The application of consensus clustering, utilizing the R package ConsensusClusterPlus (v4.2.0), facilitated the identification of distinct disease subtypes of endometriosis, specifically in relation to the hub gene associated with pyroptosis.

### 2.7. Analysis of immune cell infiltration

Endometriosis samples were analyzed for immune cell infiltration using Cell-type Identification By Estimating Relative Subsets Of RNA Transcripts (CIBERSORT) (LM22 signature matrix, p < 0.05) and the Estimation of STromal and Immune cells in MAlignant Tumor tissues (ESTIMATE) algorithms to generate immune cell abundance, Stromal Scores, and Immune Scores. Differential infiltration between endometriosis and normal samples was assessed and visualized with ggplot2 (R). Correlations between pyroptosis-related hub gene expression and LM22-defined immune cell abundance were visualized using pheatmap (R).

### 2.8. In vitro validation by quantitative real-time polymerase chain reaction (qRT-PCR)

Normal endometrial tissue samples were obtained from 10 patients without endometriosis (control group), whose normal endometrial tissue was confirmed by histopathological examination. These control samples were mostly collected from patients undergoing hysterectomy for other benign diseases (e.g., cervical intraepithelial neoplasia, cervical cancer, uterine fibroids) during the same period. Ectopic endometrial tissue samples were obtained from 10 patients with histologically confirmed endometriosis. All tissue samples were collected during surgery, immediately snap‑frozen in liquid nitrogen, and stored at −80°C until RNA extraction. Total RNA was extracted using TRIzol reagent (Invitrogen), and cDNA was synthesized with PrimeScript RT Master Mix (Takara). RT-qPCR was performed on a QuantStudio 5 system (Applied Biosystems) using SYBR Green reagents. Gene-specific primers for *KIF13B, BAG6, MYO5A, HEATR2, IL17A, TRAF6, HMGB1, AGER,* and *NF-κB* were designed (Table 1). Data were normalized to β-actin and analyzed via the 2^−ΔΔCt^ method. Statistical analyses were performed using GraphPad Prism 10.1.2. Data normality was confirmed by Shapiro-Wilk test (*p > 0.05*). Independent two-tailed Student’s t-tests were applied to compare expression levels between groups.

**Table 1:** Primer sequences for RT-qPCR.

Genes	F (5’ → 3’)	R (5’ → 3’)
*KIF13B*	GGCAGTGAACGAGCAAC	GCTGAGATAACCAGACCGA
*BAG6*	AAGACCTTGGACTCTCAAACTCG	CCTGGTAAATGAGCCGTTGTTTT
*MYO5A*	ATGACCCTCCTTCTCCTGT	TCTCTAAGCTGGCCTCTCA
*HEATR2*	CGCCTGAAGCTGTTCTCC	GTCCTTTGTCACCGTCTCG
*IL17A*	TACAACCGATCCACCTCACC	GGGGACAGAGTTCATGTGGT
*TRAF6*	TGCACCTTCAGTTACCGACT	TGCCTTACAGGTGCTTCAGA
*HMGB1*	GTCCATTGGTGATGTTGCGA	TCAGCCTTGACAACTCCCTT
*AGER*	GCCAGGCAATGAACAGGAAT	TCTGGCTTCCCAGGAATCTG
*NF-κB*	AGCAAATAGACGAGCTCCGA	TCGGTAAAGCTGAGTTTGCG
*β-actin*	CACCATTGGCAATGAGCGGTTC	AGGTCTTTGCGGATGTCCACGT

F, forward primer; R, reverse primer.

### 2.9. Statistical Analysis

R (v4.2.0) was used for all statistical analyses. Continuous data were presented as mean ± SD and compared using the Wilcoxon rank-sum test. Categorical data were analyzed via chi-square or Fisher’s exact tests. Spearman’s correlation assessed relationships between molecules. *p < 0.05* indicated significance.

## 3. Results

### 3.1. Flow chart

**Fig 1:**
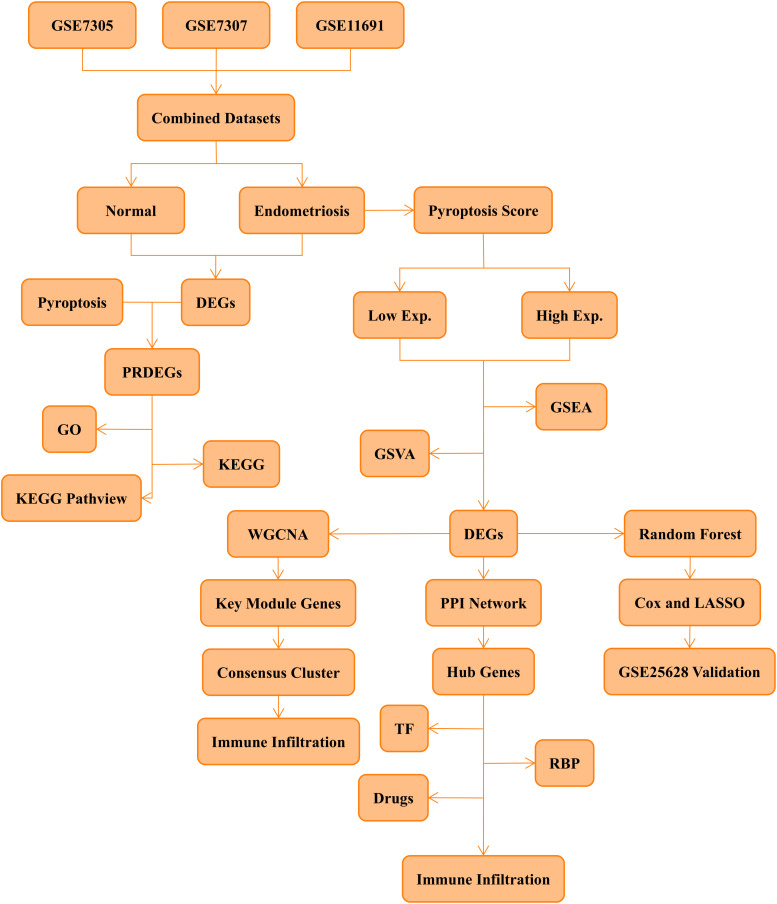
Flow chart for the comprehensive analysis of PRDEGs. (PRDEGs: Pyroptosis-related DEGs).

Flow Chart for the Comprehensive Analysis of PRDEGs in this study is shown in Fig 1.

### 3.2. Identification and characterization of PRDEGs in endometriosis

**Fig 2:**
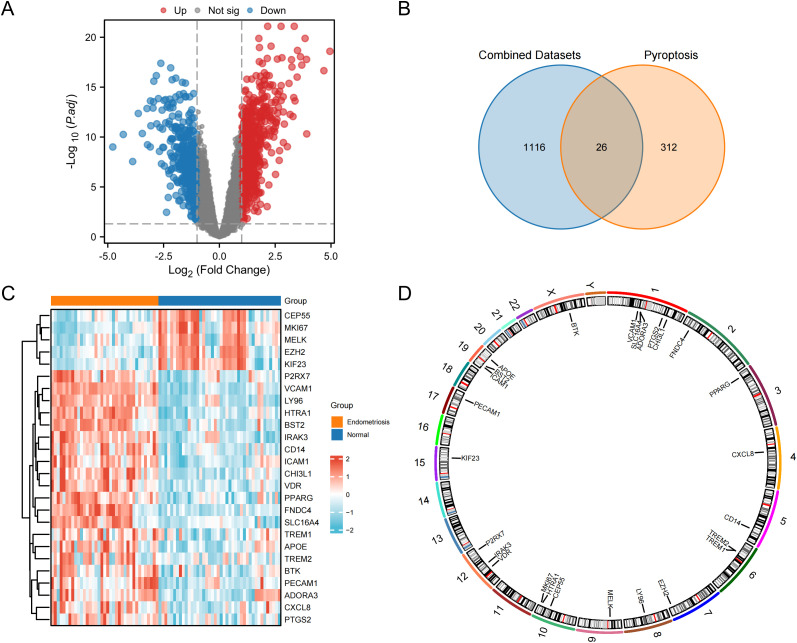
Identification and characterization of PRDEGs in EMs. **A.** Volcano plot depicting DEGs. **B.** Venn diagram illustrating the overlap between DEGs and pyroptosis-related genes associated with endometriosis. **C.** Heat map of PRDEGs in the Combined Datasets. **D.** Chromosomal localization map of PRDEGs. (PRDEGs: Pyroptosis-related DEGs; EMs: endometriosis).

A combined dataset of 37 endometriosis and 42 normal samples was obtained after batch effect correction (S1A-S1D Fig). Differential expression analysis between endometriosis and normal groups identified 1,142 DEGs (|logFC| > 1, adj.p < 0.05), including 654 up-regulated and 488 down-regulated genes (Fig 2A). Intersecting these DEGs with the curated PRGs revealed 26 PRDEGs, including *VCAM1, LY96, HTRA1, BST2, FNDC4, IRAK3, CHI3L1, PPARG, SLC16A4, VDR, CD14, ICAM1, P2RX7, MKI67, APOE, PECAM1, EZH2, ADORA3, KIF23, BTK, CXCL8, CEP55, PTGS2, MELK, TREM1,* and *TREM2* (Fig 2B). Their expression patterns and chromosomal locations are shown in Fig 2C-D.

### 3.3. Differential expression and potential diagnostic signature of PRDEGs in the combined dataset

**Fig 3:**
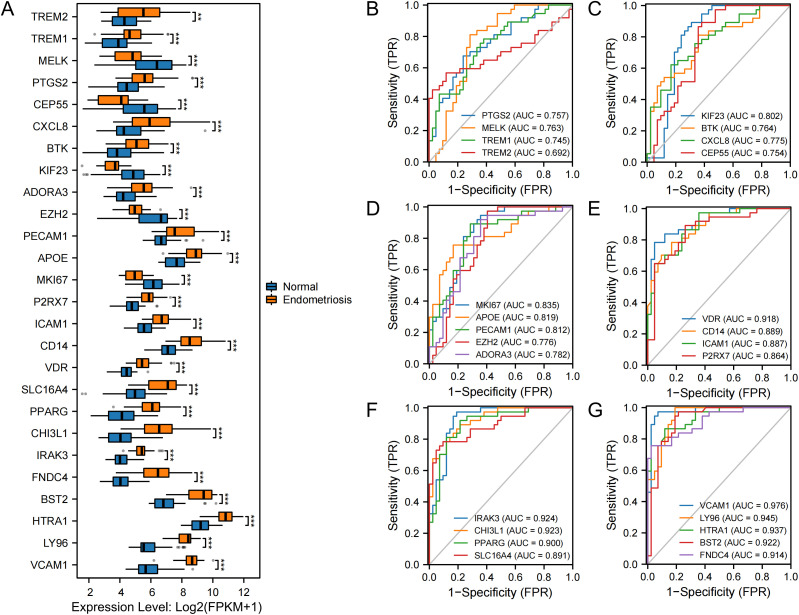
Differential expression and diagnostic potential of PRDEGs in the combined dataset. **A.** Box line plot illustrating the differential expression of PRDEGs in the Combined Dataset. **B-G.** ROC curves for 26 PRDEGs in the Combined Datasets. (PRDEGs: Pyroptosis-related DEGs).

Box plots confirmed significant expression differences of the 26 PRDEGs between endometriosis and normal groups (Fig 3A). Receiver Operating Characteristic (ROC) curve analysis showed that *VCAM1, LY96, HTRA1, BST2, FNDC4, IRAK3, CHI3L1, PPARG,* and *VDR* had high discriminatory ability with an area under the curve (AUC) greater than 0.9, suggesting their potential as candidate biomarkers for endometriosis. (Fig 3B-G). Correlation analysis revealed strong positive correlations between *MKI67* and *MELK* (r = 0.847, S2B Fig) and between *CEP55* and *MELK* (r = 0.844, S2C Fig), as well as moderate negative correlations between *MKI67* and *P2RX7* (r = −0.774, S2D Fig) and between *MKI67* and *HTRA1* (r = −0.723, S2E Fig).

### 3.4. GO and KEGG enrichment analysis of PRDEGs

**Fig 4:**
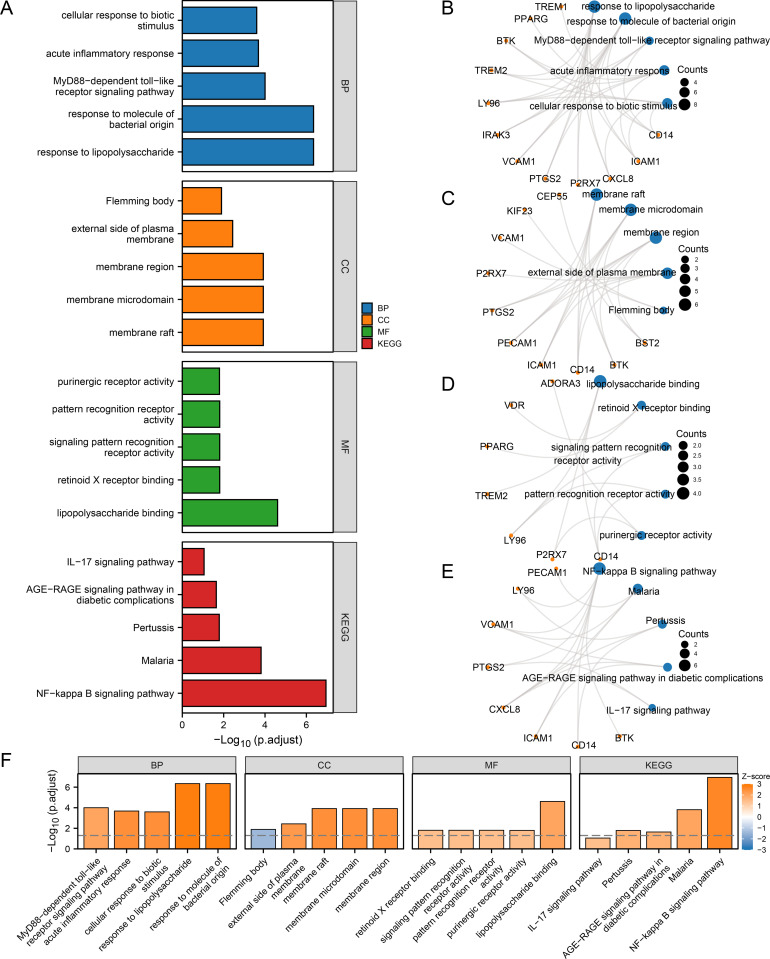
GO and KEGG enrichment analysis of PRDEGs. **A.** Histogram of GO and KEGG enrichment analysis of PRDEGs. **B-E.** Network diagrams of GO and KEGG enrichment analysis. Blue nodes represent entries, orange nodes represent molecules, and connecting lines represent the relationship between terms/pathways and genes. **F.** Combined logFC histogram. (PRDEGs: Pyroptosis-related DEGs; GO: Gene Ontology; KEGG: Kyoto Encyclopedia of Genes and Genomes).

The 26 PRDEGs were significantly enriched in specific biological functions and pathways (Fig 4A and S2 Table). The relationships between enriched GO terms and KEGG pathways are shown in Fig 4B-E. A combined logFC analysis indicated that the NF-κB signaling pathway was the most significantly up-regulated, while the Flemming body was the most prominently down-regulated (Fig 4F). Key enriched KEGG pathways included the IL-17 signaling pathway, Pertussis, Malaria, and the NF-κB signaling pathway (S3A-S3D Fig).

### 3.5. Differential gene expression analysis based on pyroptosis score

**Fig 5:**
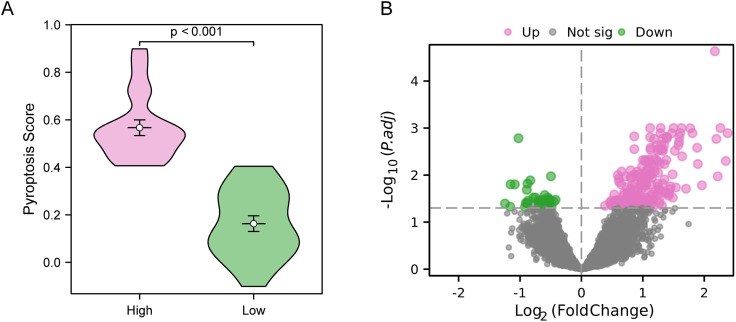
Differential gene expression analysis based on pyroptosis score. **A.** Violin plot illustrating the distribution of pyroptosis scores in the subgroups. **B.** Volcano plot depicting DEGs.

Based on the expression of 26 PRDEGs in endometriosis samples, a pyroptosis score was calculated for each sample using the ssGSEA algorithm. Patients in the combined dataset were then stratified into high and low pyroptosis expression groups according to the median score. A significant difference in score distribution was observed between the two groups (Fig 5A, *p < 0.001*), indicating that the score effectively captures the stratification of pyroptosis-related expression characteristics within the samples. Based on this stratification, differential expression analysis was performed, identifying a total of 238 DEGs between the high and low pyroptosis groups, including 203 upregulated and 35 downregulated genes (Fig 5B).

### 3.6. Development and evaluation of a gene signature for endometriosis based on DEGs

**Fig 6:**
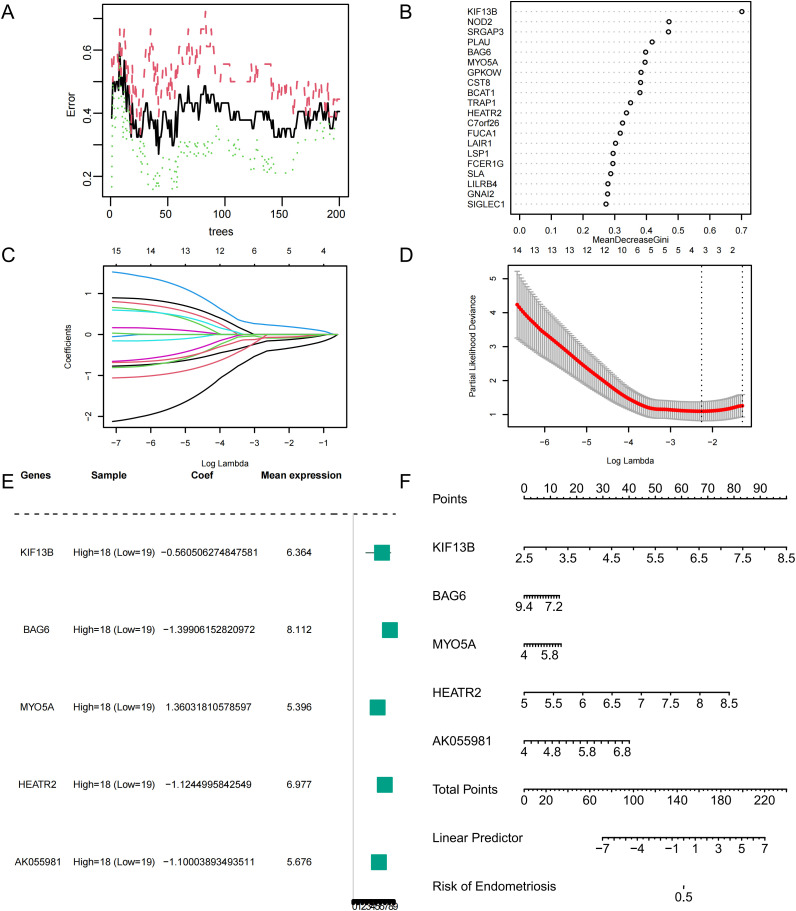
Random forest, Cox and LASSO regression analysis of the diagnostic model. **A.** Plot of the training error for the Random Forest algorithm during model development. **B.** Top 20 DEGs identified by the Random Forest model. **C.** Diagnostic model plot based on 15 DEGs identified through multifactorial Cox regression analysis. **D.** Trajectory plots illustrating the prognostic value of the 15 DEGs identified through multifactorial Cox regression analysis. **E.** Forest plot depicting the hazard ratios and confidence intervals for the 5 DEGs selected by LASSO regression analysis. (LASSO: Least Absolute Shrinkage and Selection Operator).

Random forest and LASSO regression analyses of the 238 DEGs identified five candidate genes: *KIF13B, BAG6, MYO5A, HEATR2,* and *AK055981* (Fig 6A-F). The model constructed based on these genes demonstrated a certain discriminatory ability in an independent dataset (S4A-S4D Fig), suggesting its potential reference value for the molecular identification of endometriosis. Given the limitations of sample size and variable dimensionality, the results of this model are more suitable as exploratory findings to provide candidate clues for subsequent validation.

### 3.7. GSEA and GSVA of endometriosis-related genes in the combined dataset

**Fig 7:**
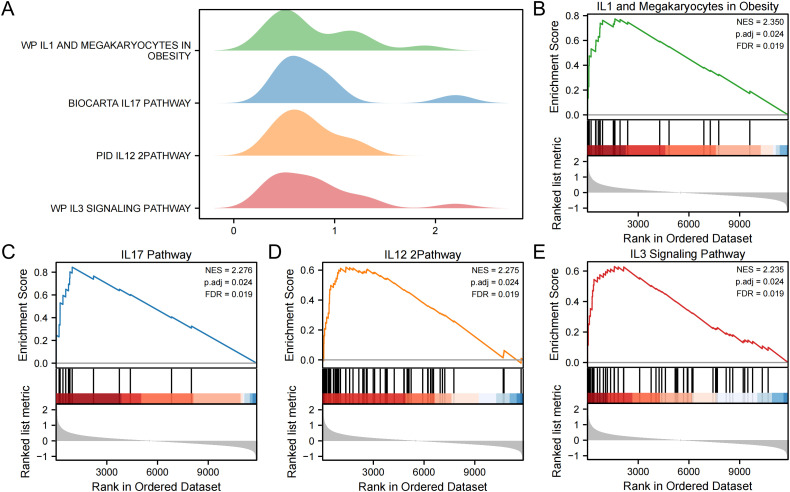
GSEA of endometriosis-related genes in the combined dataset. **A.** Enrichment plots for the top 4 biological functions enriched in the endometriosis group of the combined dataset. B-E. GSEA enrichment plots demonstrating the significant impact of endometriosis on the following pathways: IL1 and Megakaryocytes in Obesity **(B)**, IL17 Pathway **(C)**, IL12 Pathway **(D)** and IL3 Signaling Pathway **(E)**. (GSEA: Gene Set Enrichment Analysis).

Gene expression changes in the endometriosis group were significantly associated with several key biological functions and signaling pathways, including those related to IL1 and megakaryocytes in obesity, the IL17 pathway, the IL12 pathway, and the IL3 signaling pathway (Fig 7A-E and S3 Table). Furthermore, a total of 15 hallmark pathways were significantly enriched in either the high or low pyroptosis expression group (p < 0.05, S5A-S5B Fig and S4 Table). These findings indicate that pyroptosis‑related transcriptional alterations in endometriosis are closely linked to inflammatory and immune signaling pathways, particularly those involving IL-17 and IL-12.

### 3.8. WGCNA and identification of KMGs in endometriosis subgroups

**Fig 8:**
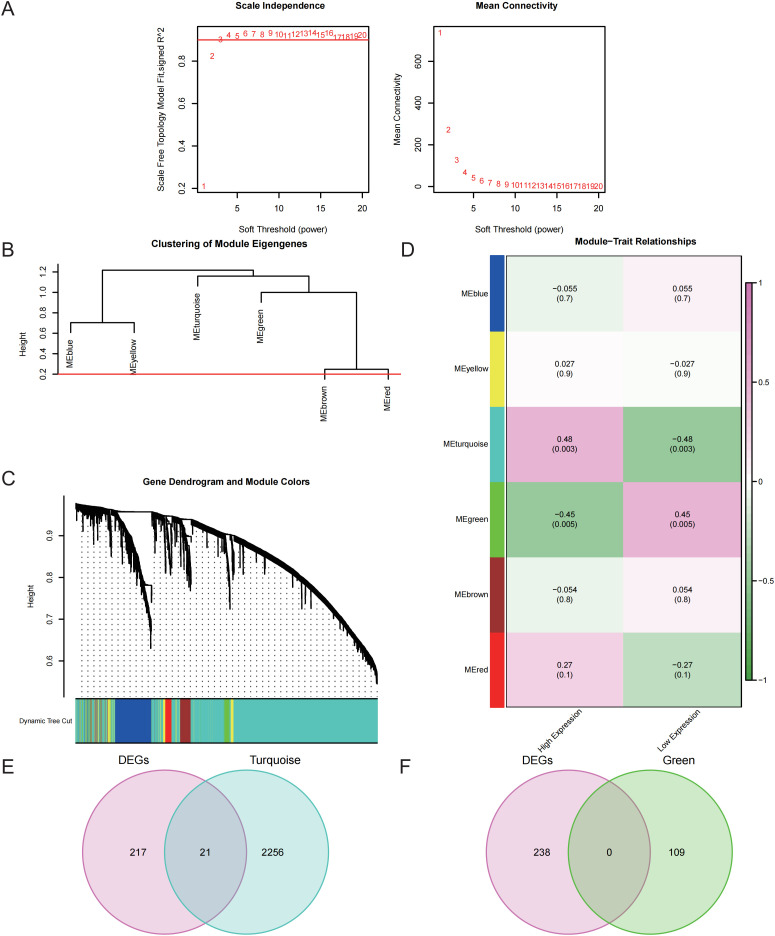
WGCNA and identification of KMGs. **A.** Scale-free network analysis for determining the optimal soft thresholding power in WGCNA. The left panel shows the scale-free fit index, and the right panel depicts the mean network connectivity. **B.** Dendrogram illustrating the hierarchical clustering of gene co-expression modules for genes exhibiting the top 25% variance. **C.** Module assignment for genes within the top 25% variance range. The upper panel shows the hierarchical clustering dendrogram, and the lower panel displays the corresponding module colors assigned to each gene. **D.** Correlation analysis between module eigengenes and the high and low pyroptosis expression groups. E-F. Venn diagrams depicting the overlap between DEGs and genes within the: **(E)** MEturquoise module, **(F)** MEgreen module. (WGCNA: Weighted Gene Co-expression Network Analysis; KMGs: Key Module Genes).

WGCNA identified six co-expression modules (MEblue, MEyellow, MEturquoise, MEgreen, MEbrown, and MEred) in the endometriosis group (Fig 8A-C). MEturquoise (|r| = 0.48) and MEgreen (|r| = 0.45) showed the strongest correlations with pyroptosis expression (Fig 8D). Intersection of DEGs from high/low pyroptosis groups with these module genes identified 21 key module genes: *NOD2, IL32, AGRP, ASGR2, BCAT1, COL11A1, SLC12A8, TLR8, MGAT4A, NABP1, SERPINE1, SAMHD1, GDF2, KIF13B, NEUROD4, CST8, NLGN4Y, UBASH3A, TNF, HSD11B2,* and *LOC100131510* (Fig 8E-F).

### 3.9. PPI network analysis and identification of Hub genes

**Fig 9:**
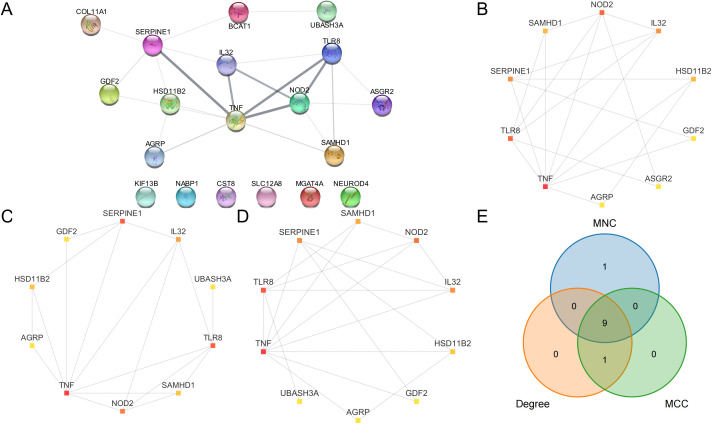
Network analysis and identification of hub genes. **A.** PPI network of KMGs. **B.** Top 10 KMGs ranked by MNC algorithm. **C.** Top 10 KMGs ranked by Degree algorithm. **D.** Top 10 KMGs ranked by MCC algorithm. **E.** Venn diagram illustrating the overlap among the top 10 KMGs identified by the MNC, Degree, and MCC algorithms. Node colors represent the ranking of each gene, with red indicating the highest rank and yellow indicating the lowest rank.

A protein-protein interaction network revealed 13 interacting KMGs (Fig 9A). From this network, nine pyroptosis-related hub genes were identified: *TNF, NOD2, TLR8, IL32, SERPINE1, SAMHD1, HSD11B2, GDF2,* and *AGRP* (Fig 9B-E). A ceRNA network involving 6 hub genes, 28 miRNAs, and 44 circRNAs was identified (S6A Fig and S5 Table). Additionally, a TF-mRNA network consisted of 57 TFs and 9 hub genes (S6B Fig and S6 Table); 21 RNA-binding proteins (RBPs) and the same 9 hub genes formed an mRNA-RBP network (S6C Fig and S7 Table); and 37 drugs or compounds were found to target the 9 hub genes in a drug-mRNA network (S6D Fig and S8 Table).

### 3.10. Immune infiltration landscape in endometriosis

**Fig 10:**
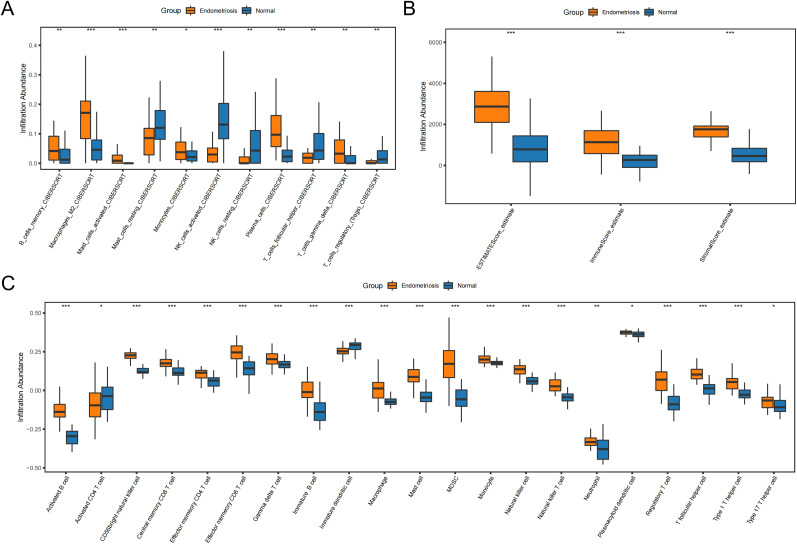
Immune infiltration landscape in endometriosis. **A.** Box plots comparing the abundance of immune cell subtypes identified by CIBERSORT between endometriosis and normal subgroups. **B.** Box plots comparing the ESTIMATE scores (Estimate Score, Immune Score, and Stromal Score) between endometriosis and normal subgroups. **c.** Box plots comparing the enrichment scores of immune cell types identified by ssGSEA between endometriosis and normal subgroups.

Significant differences in the composition of 11 immune cell types were observed between the endometriosis and normal subgroups, with macrophages M2, activated mast cells, activated NK cells, and plasma cells exhibiting particularly pronounced differences (p < 0.001, Fig 10A). The Estimate Score, Immune Score, and Stromal Score were also significantly different between the endometriosis and normal samples (p < 0.001, Fig 10B). Furthermore, 21 immune cell types showed significantly different enrichment scores between the two subgroups (p < 0.05, Fig 10C).

### 3.11. Identification of endometriosis subtypes based on KMGs expression

**Fig 11:**
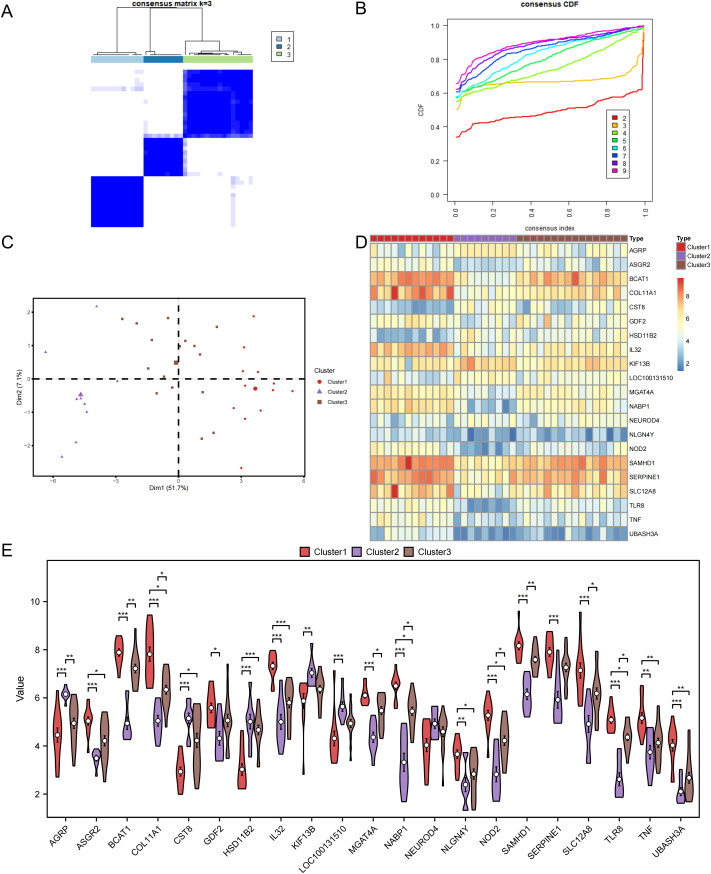
Identification of endometriosis subtypes based on KMG expression. **A.** Consensus clustering matrix for endometriosis samples, demonstrating the stability of the identified subtypes. **B.** Consistent Cumulative Distribution Function (CDF) plot for the consensus clustering analysis, indicating the optimal number of clusters. **C.** PCA plot visualizing the separation of the three identified endometriosis subtypes. **D.** Heatmap depicting the expression levels of KMGs across the three endometriosis subtypes. **E.** Violin plots of KMG expression levels within each of the three endometriosis subtypes. (KMG: Key Module Gene).

Using the expression profiles of the 21 KMGs, the endometriosis samples were classified into three stratified subgroups, which exhibited certain differences in transcriptomic expression patterns and immune infiltration characteristics (Fig 11A-B). Principal component analysis (PCA) plots confirmed that these three subtypes exhibited distinct gene expression patterns (Fig 11C). Heatmap and violin plots visualized differential KMG expression across the clusters (Fig 11D-E, *p < 0.05*). These results suggest a certain degree of heterogeneity in endometriosis at the pyroptosis-related molecular level. Given the lack of corresponding clinical staging, symptom severity, and treatment response information in the public dataset, the current clustering results are primarily used to describe differences in molecular expression characteristics, and their clinical relevance requires further investigation.

### 3.12. Correlation analysis of immune cell infiltration and KMG expression in endometriosis subtypes

**Fig 12:**
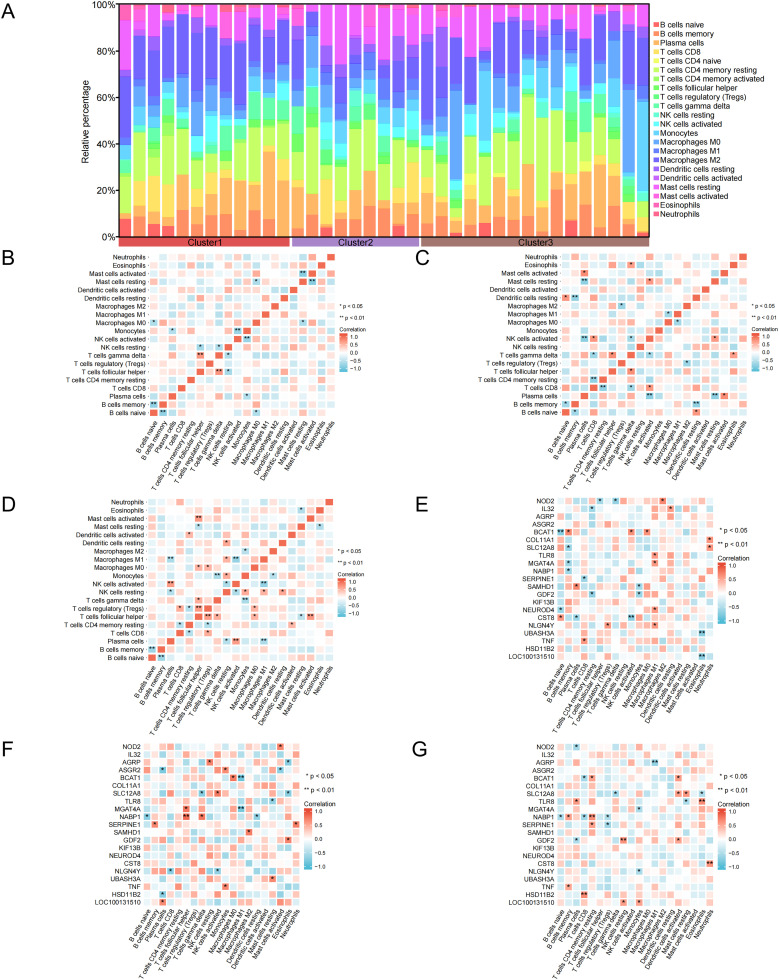
Consensus clustering immune infiltration analysis by CIBERSORT algorithm. **A.** Histogram of the percentage of immune cells. B-D. Correlation analysis of the abundance of immune cell infiltration in Endometriosis Cluster 1 **(B)**, Cluster 2 **(C)** and Cluster 3 **(D)**. E-G. Correlation of the abundance of immune cell infiltration in Endometriosis Cluster 1 **(E)**, Cluster 2 **(F)** and Cluster 3 **(G)**. Heat map of the correlation between the abundance of immune cell infiltration and KMGs. Cluster 1 in red, Cluster 2 in purple and Cluster 3 in brown. (CIBERSORT: Cell type Identification By Estimating Relative Subsets Of RNA Transcripts).

The proportion of immune cell types varied across the three clusters (Fig 12A). Distinct patterns of immune cell infiltration were strongly correlated with the specific subtypes (Fig 12B-D). In Cluster 1 (Fig 12B), T follicular helper cells and gamma delta T cells exhibited the strongest positive correlation, while activated mast cells and resting mast cells showed a strong negative correlation. In Cluster 2 (Fig 12C), activated mast cells and plasma cells demonstrated the strongest positive correlation, whereas resting dendritic cells and memory B cells exhibited the strongest negative correlation. In Cluster 3 (Fig 12D), activated mast cells and T follicular helper cells showed the strongest positive correlation, while M1 macrophages and activated NK cells displayed the most pronounced negative correlation.

Furthermore, specific KMGs were significantly correlated with immune cell infiltration levels within each subtype (Fig 12E-G). In Cluster 1, M1 macrophages demonstrated the strongest positive correlation with KMGs (Fig 12E). In Cluster 2, T follicular helper cell infiltration exhibited the strongest positive correlation with the expression of NABP1 (Fig 12F). In Cluster 3, M1 macrophages demonstrated the strongest negative correlation with AGRP (Fig 12G).

These findings indicate that each molecular subtype of endometriosis is associated with a distinct immune cell infiltration profile, and specific KMGs may play subtype-dependent roles in modulating the local immune microenvironment.

### 3.13. In vitro validation of key diagnostic genes and IL-17 pathway components

**Fig 13:**
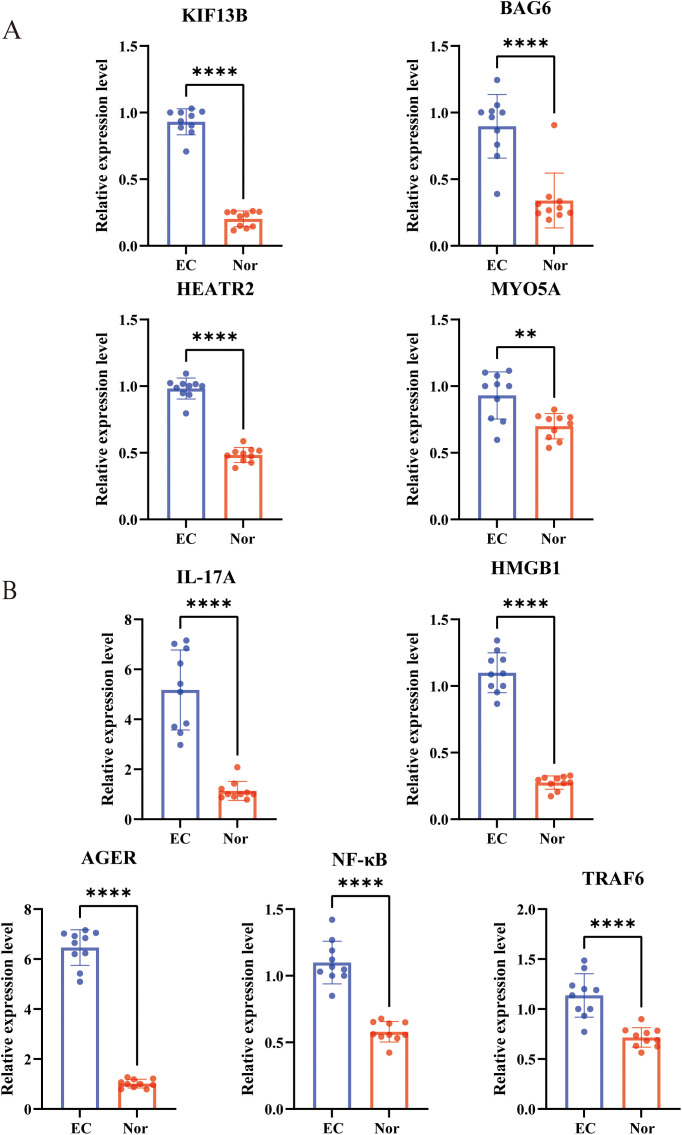
RT-qPCR analysis for diagnostic biomarkers. **A.** RT-qPCR validation of four signature genes (*KIF13B, BAG6, MYO5A,* and *HEATR2*) in patients’ ectopic (Ec) and normal (Nor) endometrial tissue samples (n = 10 per group). **B.** RT-qPCR validation of IL-17 pathway components *(IL17A, TRAF6, HMGB1, AGER,* and *NF-κB*) in the same tissue samples. (**p < 0.01, ***p < 0.001, ****p < 0.0001).

Using normal and ectopic endometrial tissue samples (n = 10 per group), RT-qPCR validation demonstrated that all four candidate signature genes (*KIF13B, BAG6, MYO5A,* and *HEATR2*) were significantly upregulated in ectopic tissues compared to eutopic controls (*p < 0.01*, Fig 13A). Similarly, IL-17 pathway components (*IL17A, TRAF6, HMGB1, AGER,* and *NF-κB*) exhibited elevated expression in ectopic tissues (*p < 0.01*, Fig 13B). These experimental results aligned with the transcriptomic data, supporting the role of PRGs and IL-17-driven inflammation in endometriosis pathogenesis.

## 4. Discussion

Endometriosis is a chronic inflammatory disease characterized by ectopic endometrial growth, leading to pelvic pain and infertility [9,10]. Although its pathogenesis remains incompletely understood, emerging evidence implicates inflammatory pathways [11,12] and pyroptosis [13,14]. Pyroptosis has been increasingly recognized as an important mediator in various diseases, including cancer and autoimmune disorders [15–18]. The presence of 26 PRDEGs suggests that pyroptosis may actively contribute to disease pathophysiology rather than being a mere bystander effect [19,20]. Previous research has established that cytokines such as IL-1β and IL-18 are key mediators in EMs [21,22]. Because the maturation and release of these cytokines are directly governed by the pyroptotic cascade, it is plausible that the ectopic endometrial microenvironment is intrinsically primed for pyroptotic cell death, which in turn fuels the continuous release of pro-inflammatory signals.

Several of the identified pyroptosis-related genes, particularly *VCAM1* and *LY96,* are involved in immune cell adhesion and innate immune recognition [23,24]. Their dysregulation in endometriosis points to a feed-forward mechanism: pyroptotic events in ectopic lesions may upregulate adhesion molecules, thereby recruiting peripheral immune cells into the peritoneal cavity and exacerbating local inflammation. The concurrent activation of the NF-κB and IL-17 signaling pathways, observed in our enrichment analyses, suggests a synergistic role in amplifying inflammatory responses. NF-κB, a master transcriptional regulator of inflammation [25], primes inflammasome components required for pyroptosis [26,27], whereas IL-17 may cooperate with pyroptotic signals to hyperactivate NF-κB. Such a vicious cycle could impede tissue resolution and promote fibrosis, offering a mechanistic explanation for the persistence of endometriotic lesions.

The candidate gene signature shows a potential association with molecular heterogeneity in endometriosis. However, it is important to emphasize that the pyroptosis score was used solely for internal expression stratification of samples; the observed consistency reflects stratification within the same scoring framework and should not be equated with independent predictive performance. Moreover, the model was built on a relatively limited sample size with a large number of candidate variables. Consequently, the current findings are best considered exploratory. Future evaluation of the model’s stability and generalizability will require larger cohorts and more rigorous validation strategies.

The existence of three distinct subgroups based on pyroptosis-related gene expression suggests heterogeneity in the molecular networks and immune microenvironment of endometriosis. However, given the lack of clinical phenotype information in the public dataset, the relationship between these subgroups and disease stage, symptom burden, or treatment response remains unknown. Therefore, these findings should be considered an exploratory observation, providing a basis for future studies that integrate complete clinical data.

RT-qPCR results using patient tissue samples provided partial support for the differential expression trends of certain candidate genes, offering preliminary experimental corroboration of the transcriptomic screening findings. However, this validation did not cover all genes in the signature (e.g., *AK055981* was not validated), and the sample size is still relatively small. Therefore, these results are more suitable as preliminary support for some molecular features rather than sufficient evidence to judge the diagnostic value of this gene signature. Further validation of the stability and potential utility of the gene panel should be conducted in larger, well-matched independent patient cohorts.

In conclusion, this bioinformatics-based exploration suggests a potential role for pyroptosis in endometriosis and identifies a candidate five-gene signature along with three molecular clusters that may reflect disease heterogeneity. A possible link between pyroptosis and IL-17-mediated inflammation is proposed. All findings remain exploratory and require validation in larger, well‑matched patient cohorts, as well as mechanistic studies to assess their therapeutic significance.

## 5. Conclusions

This study identified pyroptosis-related molecular features at the transcriptomic level and screened a panel of candidate genes with potential diagnostic significance in endometriosis. These findings provide new clues for understanding the mechanisms of inflammation and cell death in this disease, as well as insights into its molecular heterogeneity. However, due to the limited sample size, lack of independent validation with comprehensive clinical data, and absence of functional experiments, the current results are primarily exploratory. Therefore, the potential clinical significance of these findings warrants further validation in larger, well-characterized independent cohorts before any diagnostic or therapeutic claims can be made.

## Supporting information

S1 Table Pyroptosis-related genes. A comprehensive list of pyroptosis-related genes was compiled from the GeneCards database and the Gene Set Enrichment Analysis (GSEA) database. Following the merging and removal of duplicate entries, a final set of 338 unique pyroptosis-related genes was established.
(XLSX)

S2 Table GO KEGG results. To elucidate the biological functions and pathways associated with the 26 PRDEGs, GO and KEGG enrichment analyses were performed.
(DOCX)

S3 Table GSEA results. This analysis examined the relationship between the expression levels of all genes and curated gene sets representing BP, CC, and MF.
(DOCX)

S4 Table GSVA results. To investigate the differential enrichment of hallmark pathways between the high and low pyroptosis expression groups in endometriosis, GSVA was performed using the expression data of all genes in the combined dataset. The results of the GSVA are detailed in S4 Table.
(DOCX)

S5 Table ceRNA network. This analysis yielded a network comprising 6 pyroptosis-related hub genes, 28 miRNAs, and 44 circRNAs. The resulting ceRNA network was constructed.
(XLSX)

S6 Table TF-mRNA regulatory network. A TF-mRNA regulatory network was then constructed, encompassing 9 pyroptosis-related hub genes and 57 TFs.
(XLSX)

S7 Table mRNA-RBP regulatory network. An mRNA-RBP regulatory network was subsequently constructed, comprising 9 pyroptosis-related hub genes and 21 RBPs.
(XLSX)

S8 Table mRNA-drugs regulatory network. A drug-mRNA regulatory network was constructed, encompassing 9 pyroptosis-related hub genes and 37 drugs or molecular compounds.
(XLSX)

S1 Fig Batch effects removal of GSE7305, GSE7307 and GSE11691. A. Box line plots illustrating the distribution of gene expression values across datasets before batch effect removal. B. Box line plots illustrating the distribution of gene expression values in the combined dataset after batch effect removal. C. 3D PCA plot of the datasets before batch effect removal. D. 3D PCA plot of Combined Datasets after batch effect removal.
(PNG)

S2 Fig Correlation analysis of PRDEGs. A. Heatmap depicting the correlation between PRDEGs in the Combined Datasets. B-E. Scatter plots illustrating the correlations between: MKI67 and MELK (B), CEP55 and MELK (C), MKI67 and P2RX7 (D), and MKI67 and HTRA1 (E).
(PNG)

S3 Fig KEGG pathway visualization. A-D. Pathway map for KEGG enrichment analysis of PRDEGs: IL-17 signaling pathway (A), Pertussis (B), Malaria (C) and NF-kappa B signaling pathway (D).
(PNG)

S4 Fig Module validation and datasets validation of GSE25628. A-B: Calibration curve(A), DCA plots(B) for the diagnostic model based on 5 DEGs in the training dataset. C-D. Calibration curve(C), DCA plots(D) for the diagnostic model in the independent validation dataset GSE25628.
(PNG)

S5 Fig GSVA of hallmark pathways in endometriosis subgroups. A. Box plots illustrating the differential enrichment of hallmark pathways between the high and low pyroptosis expression groups. B. Heatmap depicting the enrichment scores of hallmark pathways in the two groups.
(PNG)

S6 Fig ceRNA and regulatory network of hub genes. A-D. ceRNA Network(A), TF-mRNA Regulatory Network(B), mRNA-RBP Regulatory Network(C), mRNA-Drugs Regulatory Network(D) of pyroptosis-related hub genes. orange oval for mRNA, green square for miRNA, blue diamond for circRNA, purple hexagon for TF, red circle for RBP, brown parallelogram for Drugs.
(PNG)
