# Supplementary material for: Identification and diagnostic potential of pyroptosis-related genes in endometriosis: A novel bioinformatics analysis and validation
Source: PLoS One. 2026 Jun 9;21(6):e0350751. doi: 10.1371/journal.pone.0350751 (PMC13249155; doi:10.1371/journal.pone.0350751)
Supplement: S2 Table — To elucidate the biological functions and pathways associated with the 26 PRDEGs, GO and KEGG enrichment analyses were performed. (DOCX) [file pone.0350751.s002.docx]

| ONTOLOGY | ID | Description | GeneRatio | BgRatio | pvalue | p.adjust | qvalue |
| --- | --- | --- | --- | --- | --- | --- | --- |
| BP | GO:0032496 | response to lipopolysaccharide | 9/26 | 330/18670 | 3.62E-10 | 4.50E-07 | 2.49E-07 |
| BP | GO:0002237 | response to molecule of bacterial origin | 9/26 | 343/18670 | 5.10E-10 | 4.50E-07 | 2.49E-07 |
| BP | GO:0002755 | MyD88-dependent toll-like receptor signaling pathway | 4/26 | 36/18670 | 1.69E-07 | 9.95E-05 | 5.49E-05 |
| BP | GO:0002526 | acute inflammatory response | 6/26 | 220/18670 | 4.73E-07 | 2.09E-04 | 1.15E-04 |
| BP | GO:0071216 | cellular response to biotic stimulus | 6/26 | 236/18670 | 7.13E-07 | 2.52E-04 | 1.39E-04 |
| CC | GO:0045121 | membrane raft | 6/26 | 315/19717 | 2.79E-06 | 1.21E-04 | 8.77E-05 |
| CC | GO:0098857 | membrane microdomain | 6/26 | 316/19717 | 2.84E-06 | 1.21E-04 | 8.77E-05 |
| CC | GO:0098589 | membrane region | 6/26 | 328/19717 | 3.52E-06 | 1.21E-04 | 8.77E-05 |
| CC | GO:0009897 | external side of plasma membrane | 5/26 | 393/19717 | 1.43E-04 | 3.68E-03 | 2.67E-03 |
| CC | GO:0090543 | Flemming body | 2/26 | 28/19717 | 6.19E-04 | 1.27E-02 | 9.25E-03 |
| MF | GO:0001530 | lipopolysaccharide binding | 4/25 | 35/17697 | 1.57E-07 | 2.47E-05 | 1.57E-05 |
| MF | GO:0046965 | retinoid X receptor binding | 2/25 | 17/17697 | 2.57E-04 | 1.55E-02 | 9.89E-03 |
| MF | GO:0008329 | signaling pattern recognition receptor activity | 2/25 | 20/17697 | 3.58E-04 | 1.55E-02 | 9.89E-03 |
| MF | GO:0038187 | pattern recognition receptor activity | 2/25 | 21/17697 | 3.96E-04 | 1.55E-02 | 9.89E-03 |
| MF | GO:0035586 | purinergic receptor activity | 2/25 | 25/17697 | 5.63E-04 | 1.60E-02 | 1.02E-02 |
| KEGG | hsa04064 | NF-kappa B signaling pathway | 7/18 | 104/8076 | 1.36E-09 | 1.13E-07 | 9.14E-08 |
| KEGG | hsa05144 | Malaria | 4/18 | 50/8076 | 3.73E-06 | 1.55E-04 | 1.26E-04 |
| KEGG | hsa05133 | Pertussis | 3/18 | 76/8076 | 5.90E-04 | 1.63E-02 | 1.33E-02 |
| KEGG | hsa04933 | AGE-RAGE signaling pathway in diabetic complications | 3/18 | 100/8076 | 1.31E-03 | 2.27E-02 | 1.84E-02 |
| KEGG | hsa04657 | IL-17 signaling pathway | 2/18 | 94/8076 | 1.82E-02 | 8.87E-02 | 7.20E-02 |

GO，Gene Ontology；BP，Biological Process；CC，Cellular Component；MF，Molecular Function；KEGG，Kyoto Encyclopedia of Genes and Genomes。
