# Supplementary material for: Identification and diagnostic potential of pyroptosis-related genes in endometriosis: A novel bioinformatics analysis and validation
Source: PLoS One. 2026 Jun 9;21(6):e0350751. doi: 10.1371/journal.pone.0350751 (PMC13249155; doi:10.1371/journal.pone.0350751)
Supplement: S3 Table — This analysis examined the relationship between the expression levels of all genes and curated gene sets representing BP, CC, and MF. (DOCX) [file pone.0350751.s003.docx]

| ID | setSize | enrichmentScore | NES | pvalue | p.adjust | qvalues |
| --- | --- | --- | --- | --- | --- | --- |
| WP_TYROBP_CAUSAL_NETWORK | 50 | 8.45E-01 | 3.00E+00 | 1.61E-03 | 2.41E-02 | 1.88E-02 |
| WP_MICROGLIA_PATHOGEN_PHAGOCYTOSIS_PATHWAY | 38 | 8.49E-01 | 2.84E+00 | 1.67E-03 | 2.41E-02 | 1.88E-02 |
| REACTOME_INTERLEUKIN_10_SIGNALING | 43 | 7.74E-01 | 2.70E+00 | 1.59E-03 | 2.41E-02 | 1.88E-02 |
| REACTOME_IMMUNOREGULATORY_INTERACTIONS_BETWEEN_A_LYMPHOID_AND_A_NON_LYMPHOID_CELL | 106 | 6.66E-01 | 2.69E+00 | 1.52E-03 | 2.41E-02 | 1.88E-02 |
| KEGG_LEISHMANIA_INFECTION | 67 | 7.04E-01 | 2.64E+00 | 1.56E-03 | 2.41E-02 | 1.88E-02 |
| KEGG_HEMATOPOIETIC_CELL_LINEAGE | 82 | 6.77E-01 | 2.61E+00 | 1.57E-03 | 2.41E-02 | 1.88E-02 |
| REACTOME_GENERATION_OF_SECOND_MESSENGER_MOLECULES | 29 | 8.08E-01 | 2.55E+00 | 1.78E-03 | 2.41E-02 | 1.88E-02 |
| REACTOME_CHEMOKINE_RECEPTORS_BIND_CHEMOKINES | 52 | 7.16E-01 | 2.55E+00 | 1.63E-03 | 2.41E-02 | 1.88E-02 |
| WP_LUNG_FIBROSIS | 56 | 6.92E-01 | 2.52E+00 | 1.62E-03 | 2.41E-02 | 1.88E-02 |
| KEGG_SYSTEMIC_LUPUS_ERYTHEMATOSUS | 49 | 7.10E-01 | 2.51E+00 | 1.63E-03 | 2.41E-02 | 1.88E-02 |
| REACTOME_INTERLEUKIN_4_AND_INTERLEUKIN_13_SIGNALING | 103 | 6.22E-01 | 2.50E+00 | 1.51E-03 | 2.41E-02 | 1.88E-02 |
| KEGG_CHEMOKINE_SIGNALING_PATHWAY | 165 | 5.77E-01 | 2.49E+00 | 1.42E-03 | 2.41E-02 | 1.88E-02 |
| KEGG_INTESTINAL_IMMUNE_NETWORK_FOR_IGA_PRODUCTION | 41 | 7.03E-01 | 2.41E+00 | 1.63E-03 | 2.41E-02 | 1.88E-02 |
| REACTOME_NEUTROPHIL_DEGRANULATION | 402 | 5.11E-01 | 2.40E+00 | 1.23E-03 | 2.41E-02 | 1.88E-02 |
| KEGG_NOD_LIKE_RECEPTOR_SIGNALING_PATHWAY | 54 | 6.63E-01 | 2.38E+00 | 1.63E-03 | 2.41E-02 | 1.88E-02 |
| KEGG_CYTOKINE_CYTOKINE_RECEPTOR_INTERACTION | 230 | 5.34E-01 | 2.38E+00 | 1.37E-03 | 2.41E-02 | 1.88E-02 |
| WP_IL1_AND_MEGAKARYOCYTES_IN_OBESITY | 24 | 7.74E-01 | 2.35E+00 | 1.77E-03 | 2.41E-02 | 1.88E-02 |
| BIOCARTA_IL17_PATHWAY | 15 | 8.44E-01 | 2.28E+00 | 1.83E-03 | 2.41E-02 | 1.88E-02 |
| PID_IL12_2PATHWAY | 58 | 6.20E-01 | 2.28E+00 | 1.61E-03 | 2.41E-02 | 1.88E-02 |
| WP_IL3_SIGNALING_PATHWAY | 48 | 6.30E-01 | 2.23E+00 | 1.61E-03 | 2.41E-02 | 1.88E-02 |

GSEA，Gene Set Enrichment Analysis。
