# Supplementary material for: Identification and diagnostic potential of pyroptosis-related genes in endometriosis: A novel bioinformatics analysis and validation
Source: PLoS One. 2026 Jun 9;21(6):e0350751. doi: 10.1371/journal.pone.0350751 (PMC13249155; doi:10.1371/journal.pone.0350751)
Supplement: S4 Table — To investigate the differential enrichment of hallmark pathways between the high and low pyroptosis expression groups in endometriosis, GSVA was performed using the expression data of all genes in the combined dataset. The results of the GSVA are detailed in S4 Table. (DOCX) [file pone.0350751.s004.docx]

| ID | logFC | AveExpr | p value | adj.p value |
| --- | --- | --- | --- | --- |
| HALLMARK_INTERFERON_GAMMA_RESPONSE | -4.29E-01 | -2.04E-02 | 2.43E-07 | 1.18E-05 |
| HALLMARK_ALLOGRAFT_REJECTION | -4.43E-01 | -2.31E-02 | 4.70E-07 | 1.18E-05 |
| HALLMARK_COMPLEMENT | -3.64E-01 | -1.44E-02 | 3.45E-06 | 5.74E-05 |
| HALLMARK_KRAS_SIGNALING_UP | -3.55E-01 | 5.63E-03 | 6.66E-06 | 8.32E-05 |
| HALLMARK_INFLAMMATORY_RESPONSE | -3.74E-01 | -2.62E-02 | 1.08E-05 | 1.08E-04 |
| HALLMARK_IL6_JAK_STAT3_SIGNALING | -3.56E-01 | -3.45E-02 | 7.11E-05 | 5.92E-04 |
| HALLMARK_TNFA_SIGNALING_VIA_NFKB | -3.33E-01 | 1.04E-02 | 9.15E-05 | 6.54E-04 |
| HALLMARK_INTERFERON_ALPHA_RESPONSE | -3.41E-01 | -3.22E-02 | 1.78E-04 | 1.11E-03 |
| HALLMARK_APOPTOSIS | -2.69E-01 | 2.94E-02 | 4.99E-04 | 2.77E-03 |
| HALLMARK_IL2_STAT5_SIGNALING | -2.65E-01 | 6.18E-03 | 5.73E-04 | 2.86E-03 |
| HALLMARK_ANDROGEN_RESPONSE | -2.25E-01 | 1.05E-02 | 4.39E-03 | 2.00E-02 |
| HALLMARK_PEROXISOME | 1.92E-01 | 3.76E-02 | 1.78E-02 | 6.84E-02 |
| HALLMARK_P53_PATHWAY | -1.74E-01 | 1.34E-02 | 1.97E-02 | 6.84E-02 |
| HALLMARK_COAGULATION | -1.85E-01 | -1.63E-02 | 2.03E-02 | 6.84E-02 |
| HALLMARK_EPITHELIAL_MESENCHYMAL_TRANSITION | -1.99E-01 | 4.78E-03 | 2.16E-02 | 6.84E-02 |
| HALLMARK_HYPOXIA | -1.68E-01 | 1.63E-02 | 2.19E-02 | 6.84E-02 |
| HALLMARK_REACTIVE_OXYGEN_SPECIES_PATHWAY | -1.89E-01 | 4.40E-02 | 4.24E-02 | 1.25E-01 |
| HALLMARK_MYC_TARGETS_V1 | 2.14E-01 | 5.75E-02 | 4.70E-02 | 1.31E-01 |

GSVA，Gene Set Variation Analysis。
