# Supplementary figures and images for: Identification and diagnostic potential of pyroptosis-related genes in endometriosis: A novel bioinformatics analysis and validation
Source: PLoS One. 2026 Jun 9;21(6):e0350751. doi: 10.1371/journal.pone.0350751 (PMC13249155; doi:10.1371/journal.pone.0350751)

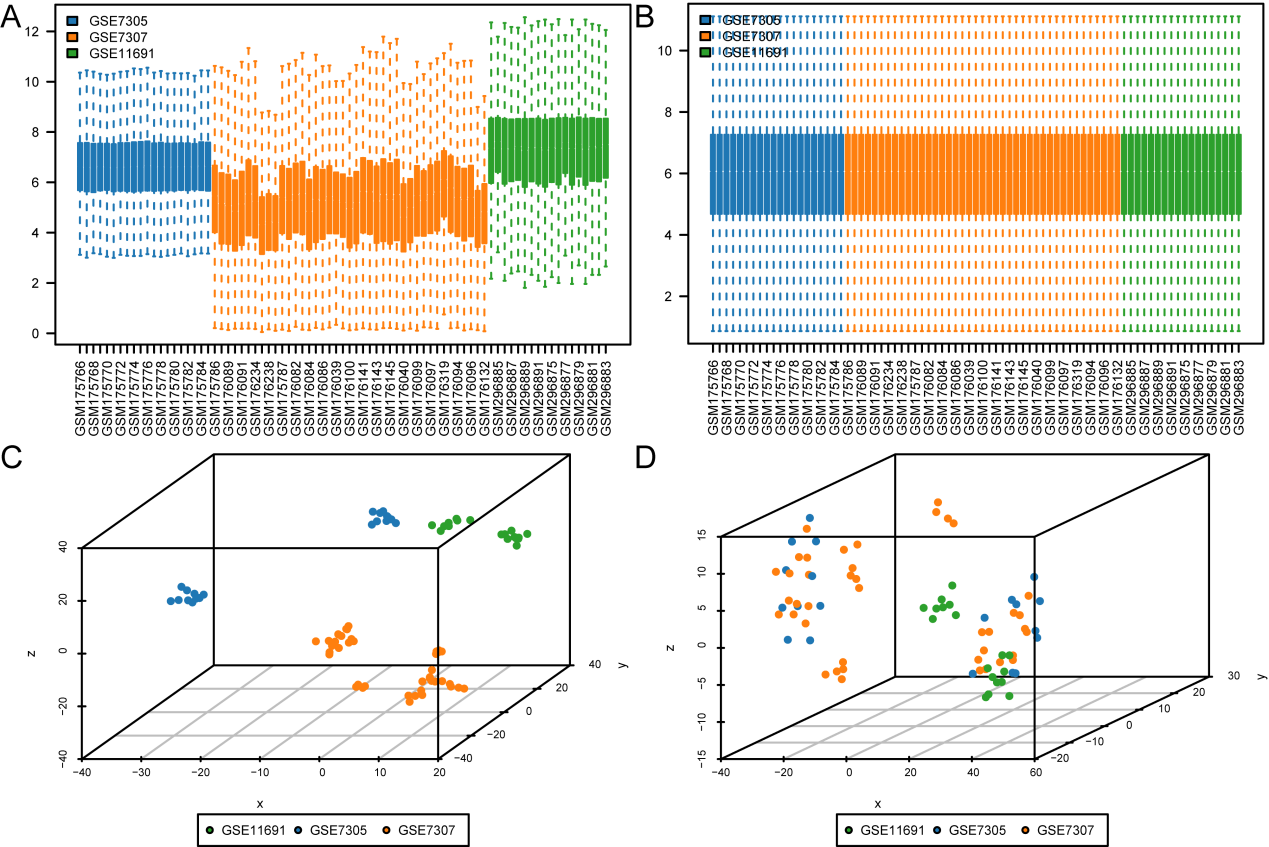

Supplement: S1 Fig — A. Box line plots illustrating the distribution of gene expression values across datasets before batch effect removal. B. Box line plots illustrating the distribution of gene expression values in the combined dataset after batch effect removal. C. 3D PCA plot of the datasets before batch effect removal. D. 3D PCA plot of Combined Datasets after batch effect removal. (PNG) [file pone.0350751.s009.png]

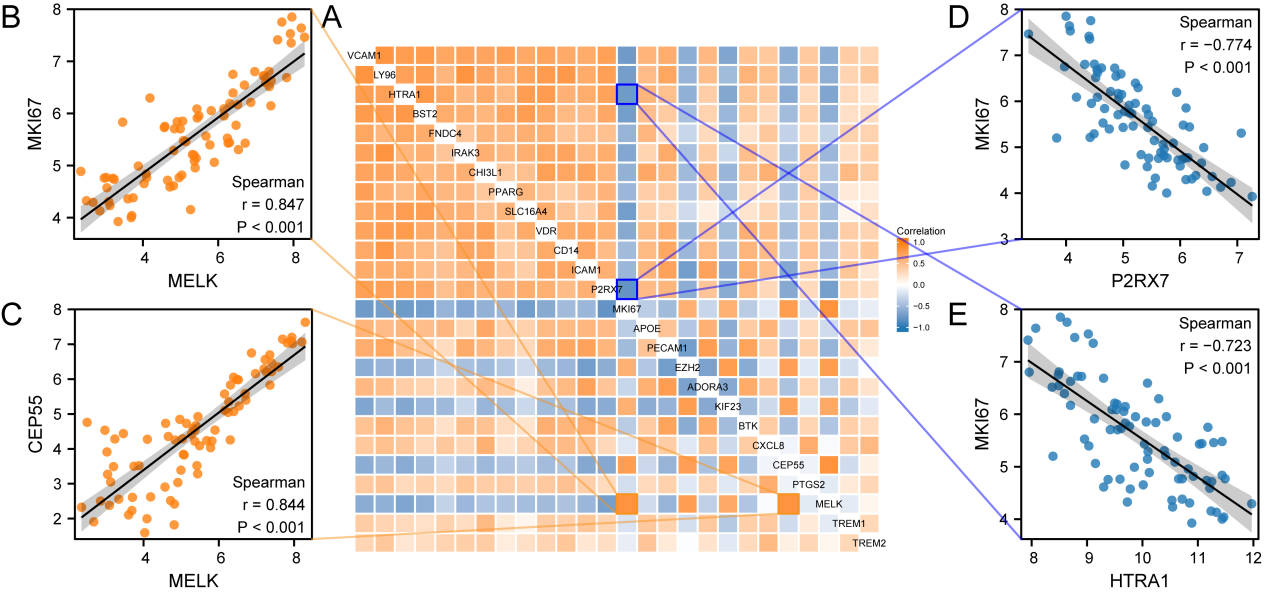

Supplement: S2 Fig — A. Heatmap depicting the correlation between PRDEGs in the Combined Datasets. B-E. Scatter plots illustrating the correlations between: MKI67 and MELK (B), CEP55 and MELK (C), MKI67 and P2RX7 (D), and MKI67 and HTRA1 (E). (PNG) [file pone.0350751.s010.png]

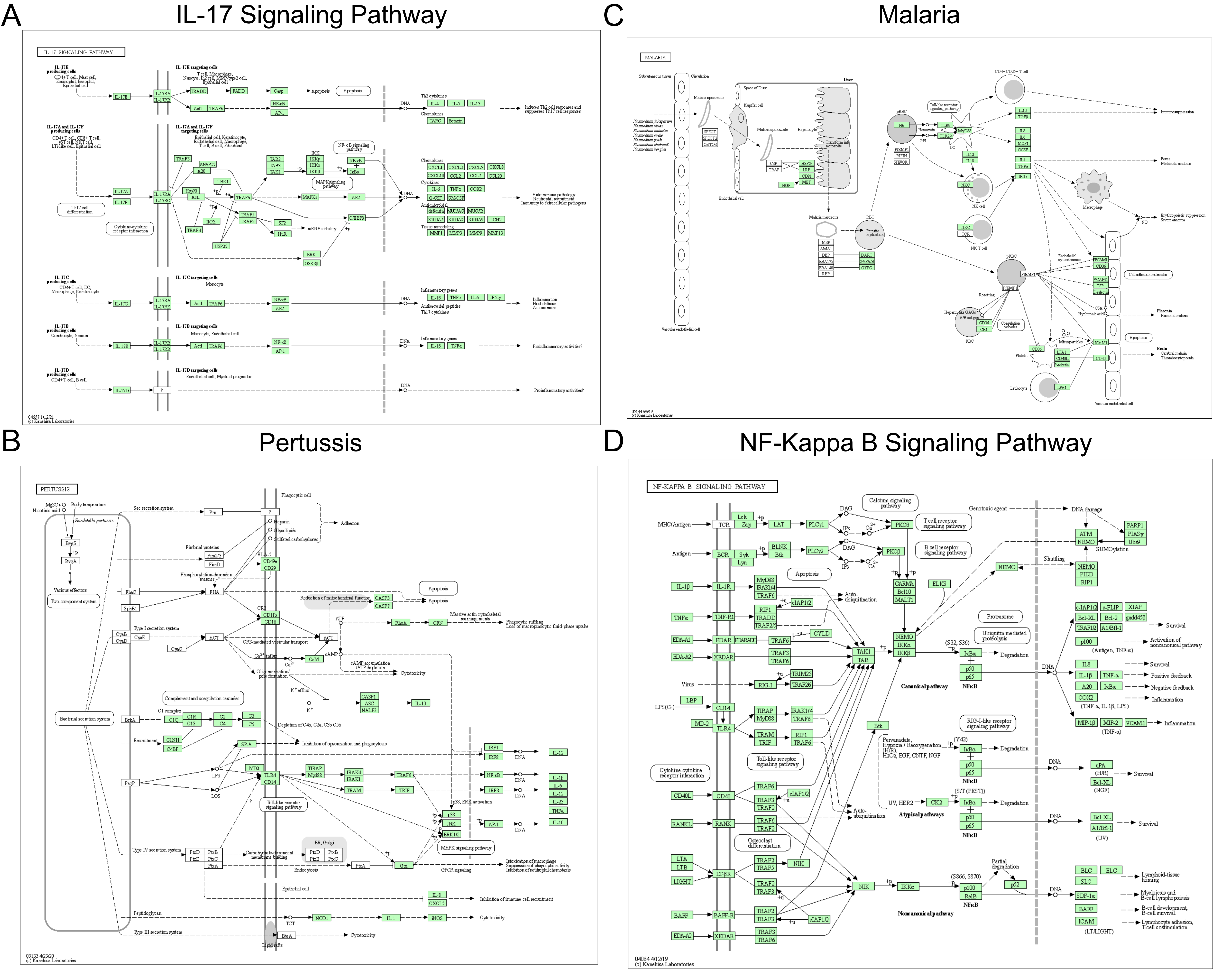

Supplement: S3 Fig — A-D. Pathway map for KEGG enrichment analysis of PRDEGs: IL-17 signaling pathway (A), Pertussis (B), Malaria (C) and NF-kappa B signaling pathway (D). (PNG) [file pone.0350751.s011.png]

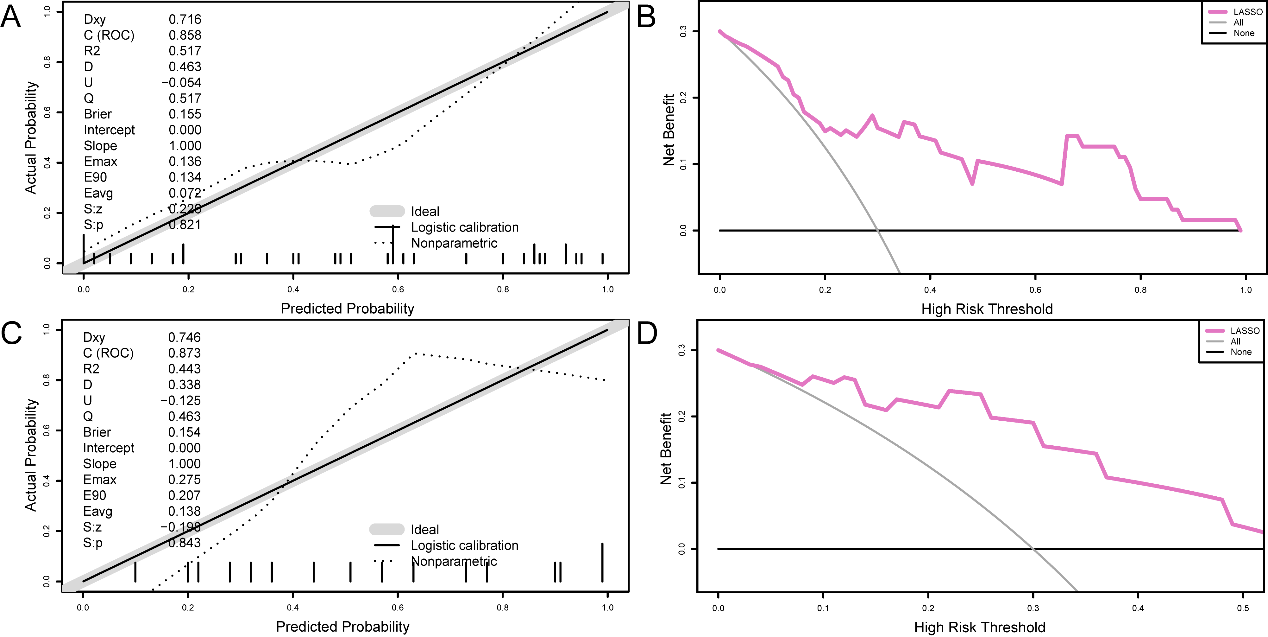

Supplement: S4 Fig — A-B: Calibration curve(A), DCA plots(B) for the diagnostic model based on 5 DEGs in the training dataset. C-D. Calibration curve(C), DCA plots(D) for the diagnostic model in the independent validation dataset GSE25628. (PNG) [file pone.0350751.s012.png]

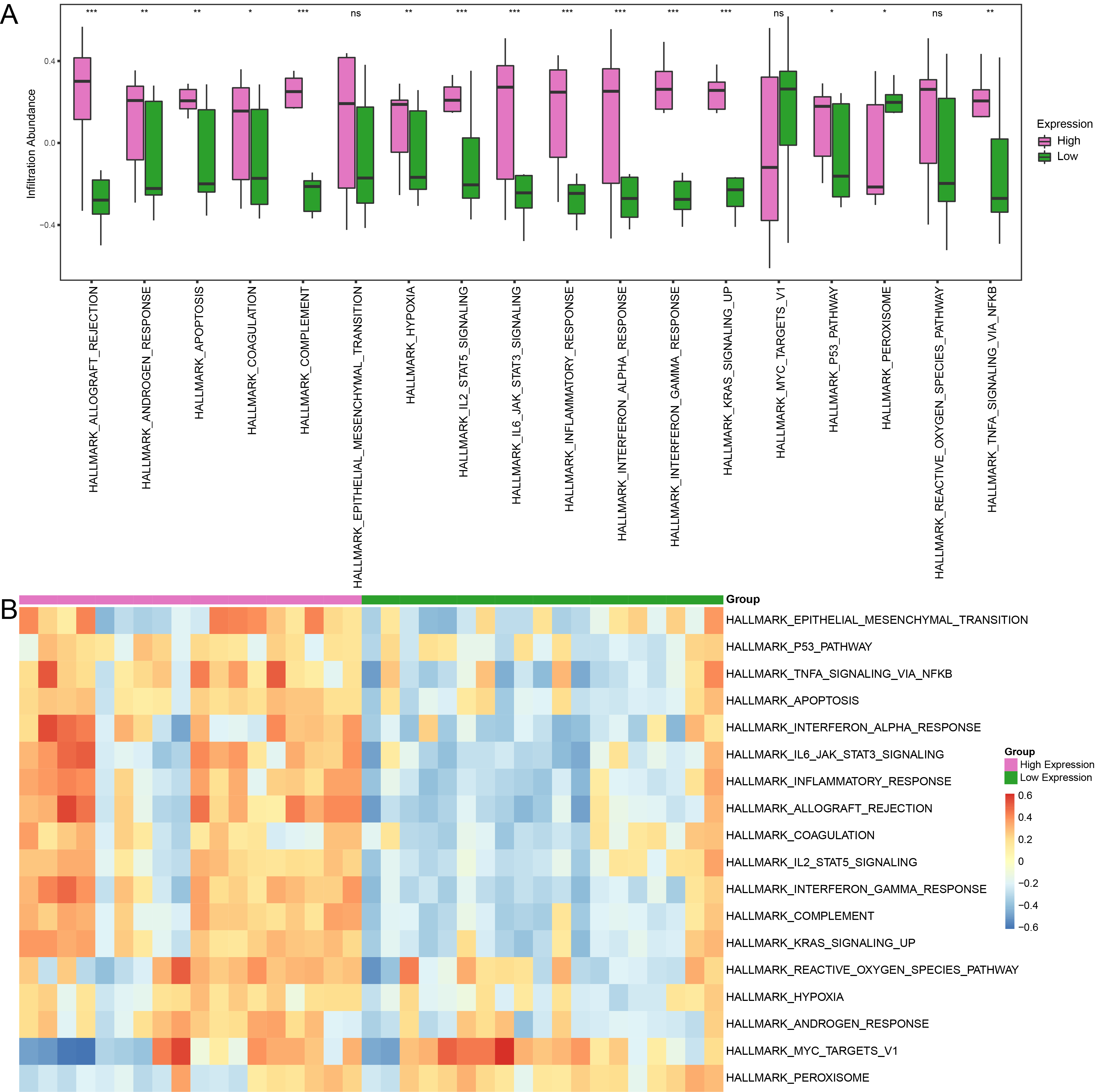

Supplement: S5 Fig — A. Box plots illustrating the differential enrichment of hallmark pathways between the high and low pyroptosis expression groups. B. Heatmap depicting the enrichment scores of hallmark pathways in the two groups. (PNG) [file pone.0350751.s013.png]

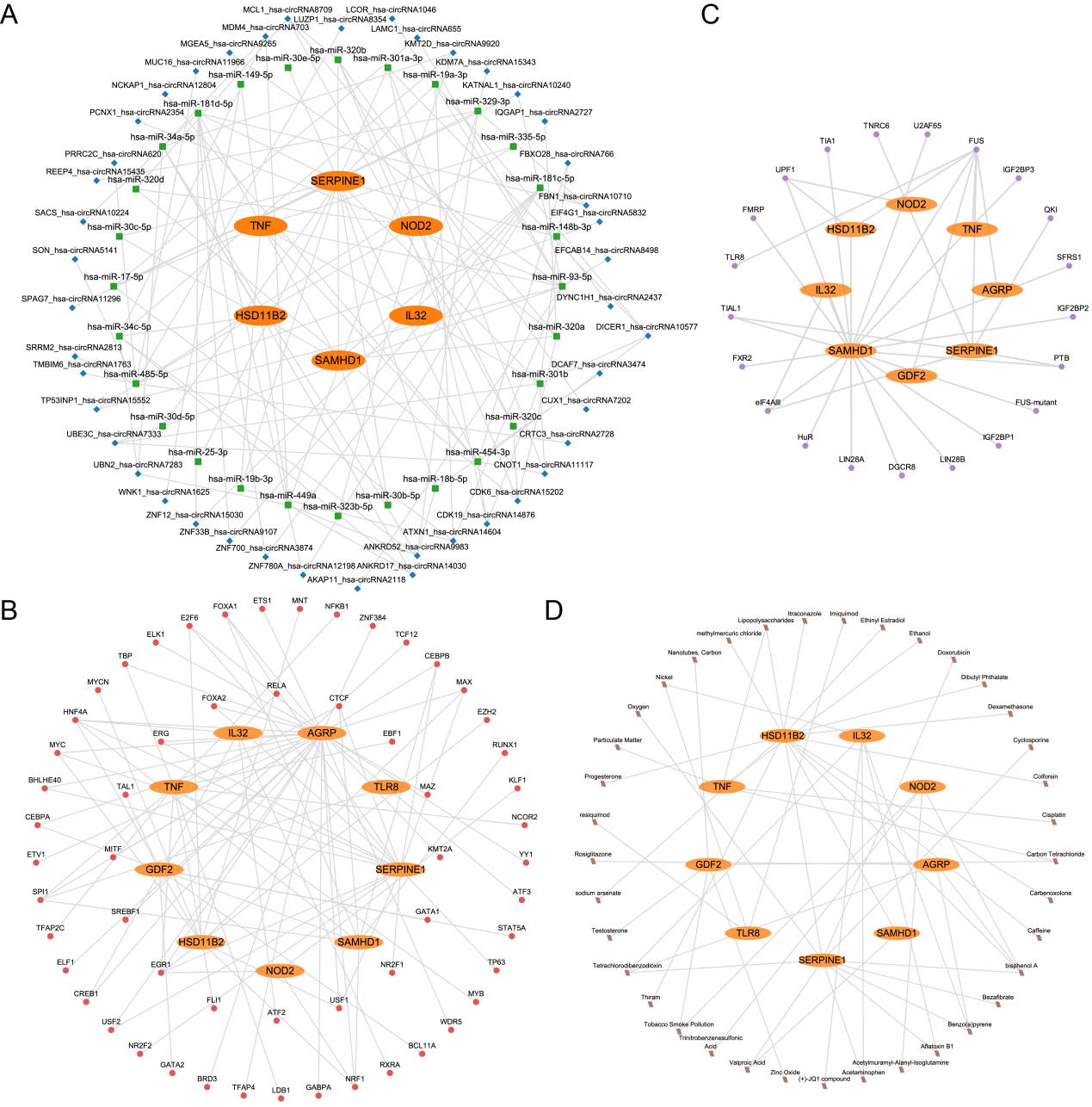

Supplement: S6 Fig — A-D. ceRNA Network(A), TF-mRNA Regulatory Network(B), mRNA-RBP Regulatory Network(C), mRNA-Drugs Regulatory Network(D) of pyroptosis-related hub genes. orange oval for mRNA, green square for miRNA, blue diamond for circRNA, purple hexagon for TF, red circle for RBP, brown parallelogram for Drugs. (PNG) [file pone.0350751.s014.png]
